# Extracellular matrix remodeling following myocardial infarction influences the therapeutic potential of mesenchymal stem cells

**DOI:** 10.1186/scrt403

**Published:** 2014-01-24

**Authors:** Kelly Elizabeth Sullivan, Kyle Patrick Quinn, Katherine Michele Tang, Irene Georgakoudi, Lauren Deems Black

**Affiliations:** 1 Department of Biomedical Engineering, Tufts University, 4 Colby Street, Medford, MA 02155, USA; 2 Cellular, Molecular and Developmental Biology Program, Sackler School of Graduate Biomedical Sciences, Tufts University School of Medicine, 145 Harrison Ave, Boston, MA 02111, USA

## Abstract

**Introduction:**

Although stem cell therapy is a promising treatment for myocardial infarction, the minimal functional improvements observed clinically limit its widespread application. A need exists to maximize the therapeutic potential of these stem cells by first understanding what factors within the infarct microenvironment affect their ability to regenerate the necrotic tissue. In this study, we assessed both differentiation capacity and paracrine signaling as a function of extracellular matrix remodeling after myocardial infarction.

**Methods:**

Mechanical and compositional changes to the decellularized infarcted myocardium were characterized to understand how the extracellular environment, specifically, was altered as a function of time after coronary artery ligation in Sprague–Dawley rats. These alterations were first modeled in a polyacrylamide gel system to understand how the variables of composition and stiffness drive mesenchymal stem cell differentiation towards a cardiac lineage. Finally, the paracrine secretome was characterized as a function of matrix remodeling through gene and protein expression and conditioned media studies.

**Results:**

The decellularized infarct tissue revealed significant alterations in both the mechanical and compositional properties of the ECM with remodeling following infarction. This altered microenvironment dynamically regulates the potential for early cardiac differentiation. Whereas Nkx2.5 expression is limited in the presence of chronic remodeled matrix of increased stiffness, GATA4 expression is enhanced. In addition, the remodeled matrix promotes the expression of several proangiogenic, prosurvival, antifibrotic, and immunomodulatory growth factors. In particular, an increase in HGF and SDF1 expression and secretion by mesenchymal stem cells can rescue oxidatively stressed cardiomyocytes *in vitro*.

**Conclusions:**

This study demonstrated that decellularization of diseased tissue allows for the exclusive analysis of the remodeled matrix and its ability to influence significantly the cellular phenotype. Characterization of cell fate as a function of myocardial remodeling following infarction is critical in developing the ideal strategy for cell implantation to maximize tissue regeneration and to ultimately reduce the prevalence and severity of heart failure.

## Introduction

The prevalence and severity of heart failure following myocardial infarction (MI) warrants the investigation into new and innovative treatment options [1]. The most commonly studied approach is stem cell therapy, which strives to regenerate the necrotic myocardium with multi- or pluripotent stem cells capable of rescuing the organ through their differentiation toward contractile cardiomyocytes or proangiogenic and prosurvival paracrine signaling to native cells of the injured heart [2-6]. However, clinical trials with unfractionated mononuclear bone marrow cells have only demonstrated the ability to promote a slight increase in contractility in those patients with a severe MI [7,8]. Identifying which variables within the infarct environment regulate their regenerative potential *in vivo* is critical in developing the ideal implantation strategy to maximize the functional benefits achieved after injection [6,9].

*In vivo* animal studies have presented conflicting evidence about the cardiac differentiation potential of MSCs within the necrotic scar and whether those cells that do integrate and express markers of a myogenic lineage contribute to functional repair [10]. Many researchers have argued that their ability to decrease infarct volume and promote contractility is most commonly through their release of soluble factors, which have demonstrated the ability to (a) promote survival of stressed and necrotic cardiomyocytes [11,12], (b) initiate angiogenesis to restore oxygen and nutrient delivery [13], (c) alter the inflammatory cascade [14], (d) assist in stem cell homing [15], and (e) limit excessive remodeling with antifibrotic factors [16]. However, the MSC secretome after implantation is poorly understood, and to harness its full potential, we must characterize what factors within the infarct microenvironment drive its expression profile [17].

The significance of the extracellular matrix (ECM) in the development and function of tissues and organ systems has been reevaluated and is now identified as a collection of signaling moieties that take part in the bidirectional exchange between the intracellular and extracellular environments [18]. Therefore, recent studies have reconsidered the role of cellular and ECM interactions and the critical functions that these interactions have throughout development, native tissue function, and disease progression [19-21]. 

Research has demonstrated that the differentiation potential of MSCs is regulated by both substrate composition [22] and stiffness [23]. However, these studies have identified only the independent effects of these two variables, but given the crosstalk between composition and stiffness [24-26], it is important to consider how they influence cells both synergistically and antagonistically. In addition, although studies have shown that both differentiation and growth-factor stimulation in the heart is integrin mediated [27], *in vitro* studies have focused on the effects of singular ECM proteins, whereas the native matrix is a complex milieu of proteins, glycoproteins, and polysaccharides [28]. With the development of decellularization techniques [29], researchers have been able to explore and predict how this dynamic network regulates cell fate *in vivo* through *in vitro* studies. For example, recent studies have demonstrated that complex cardiac ECM promotes cardiomyocyte proliferation [30], progenitor cell differentiation toward a cardiac lineage [31], and contractility of the left ventricle when injected after MI [32]. Given that the extracellular environment is significantly remodeled as a function of time after infarction, with dramatic alterations in both stiffness and composition [33], it is critical to understand how these changes affect the therapeutic potential of MSCs.

In this study, we investigated how the altered biophysical properties of the myocardium after MI affect the regenerative potential of MSCs *in vitro*. The mechanical and compositional changes to the extracellular environment were first characterized as a function of time after MI, and early and late infarct environments were recapitulated in a polyacrylamide gel system. Although the potential of MSCs for functional cardiac differentiation is questionable [34-37], our work demonstrated that both the increased stiffness and altered matrix composition of the late infarct environment severely abrogated the expression of the early cardiac transcription factor Nkx2.5. In contrast, the remodeled matrix (both composition and stiffness) enhanced the expression of another cardiac transcription factor, GATA4.

We also investigated alterations in paracrine signaling in response to infarct matrix and determined that the late, remodeled matrix significantly enhances the expression of several growth factors, including HGF (an antifibrotic and angiogenic growth factor) and SDF1 (a stem cell homing and prosurvival factor). Conditioned media from cells cultured in the presence of the remodeled matrix had the greatest potential to rescue cardiomyocytes after oxidative stress *in vitro*. We hypothesize that the enhancement of GATA4 expression observed within the late infarct environment promotes the release of beneficial soluble factors by MSCs. The goal of this study was to identify how the remodeled ECM environment after infarction affects the early cardiac differentiation potential and paracrine signaling of MSCs.

## Methods

### Characterization of extracellular environment after MI

#### Decellularization of infarcted myocardium

**Figure 1:**
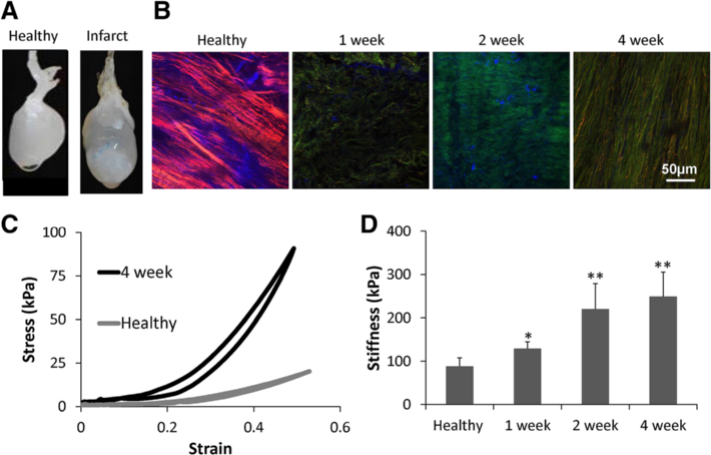
**Characterization of decellularized myocardial infarcts as a function of remodeling time. (A)** The decellularized scar appears physically distinct from the noninfarcted region of the myocardium. **(B)** Representative confocal images acquired 25 μm from tissue surface. Composite images demonstrate an increase in collagen deposition after infarct (Forward SHG in red, backward SHG in green, two-photon excited fluorescence (TPEF) emission between 500 and 550 nm in blue). **(C)** Representative stress–strain curves derived from mechanically testing decellularized myocardial strips derived from both healthy left ventricular tissue and 4-week scar tissue. **(D)** The tangent modulus of the left ventricle increases with remodeling time after myocardial infarction (*n* = 4 to 6 for each condition).

All animal experiments were performed in accordance with the US Animal Welfare Act and institutional guidelines and were approved by the Institutional Animal Care and Use Committee at Tufts University. MI was induced in male Sprague–Dawley rats (250 to 275 g) by permanently ligating the left coronary artery with a 6–0 Prolene suture. Generation of a significant infarct was verified if 40% or more of the left ventricle was blanched after artery ligation. Animals with a significant infarct were allowed to recover for 1, 2, or 4 weeks. Hearts were isolated at the respective time points and underwent retrograde perfusion decellularization with 1% sodium dodecylsulfate (SDS) by ligating the three major branches of the transverse aortic arch and advancing an 18-G cannula through the descending aorta. Decellularization was confirmed when the tissue became translucently clear, usually after 48 to 72 hours of perfusion with 3 to 6 L of 1% SDS (Figure 1A). Previous work verified that decellularization with this method is successful by the absence of cellular DNA [29].

#### Structural analysis of collagen via second harmonic generation imaging

Regions of the decellularized scar were sectioned into strips and imaged via nonlinear optical microscopy. Images were acquired on a Leica TCS SP2 confocal microscope equipped with a Ti:sapphire laser (Spectra Physics, Mountain View, CA, USA) tuned to 800 nm. By using a 63× objective (1.2 NA), image stacks (512 × 512 pixel, 238 × 238 μm^2^ field of view) were acquired at 5-μm increments from the surface of the tissue by using a set of non-descanned PMTs. Second harmonic generation (SHG) images were collected in the backward and forward direction by using 400 (±10)-nm bandpass filters. To measure collagen cross-link fluorescence, TPEF was simultaneously measured by using a 525 (±25)-nm filter. Image intensities were normalized for PMT gain and laser power, as previously described [38]. The average backward SHG, forward SHG, and TPEF intensities within the first 100 μm from the tissue surface were computed from each acquired image volume.

#### Mechanical characterization of decellularized infarcted myocardium

Because of the nondestructive nature of the optical-imaging protocol, the mechanical properties of the imaged samples were also analyzed by using a previously described setup [39]. A custom-built imaging-based device was used to measure the thickness of the tissue samples, whereas Vernier calipers measured both the width and length of the tissue. These measurements were used to calculate the initial cross-sectional area of the sample to convert force values to stress measurements. Excess moisture was removed from the samples before they were mounted on two footplates with cyanoacrylate. The sample was submerged in a bath of 1× PBS, and the plates were carefully aligned onto a custom-built uniaxial mechanical stretcher. One foot was held in a fixed position, whereas the other was connected to a lever arm with the capacity to measure and control both displacement and force (model 400B; Aurora, Ontario, Canada). All measurements were made in the circumferential direction of the heart, as this is the overall average alignment of the ECM in the ventricular wall [40]. Samples were preconditioned with 10 cycles of quasi-static (45 mm/min) displacement to 60% strain. Samples were allowed 2 minutes of viscoelastic recovery in an unloaded configuration and then exposed to 100% strain for six cycles. The sixth cycle was analyzed, and the tangential modulus was calculated in the linear region of the stress–strain curve, which was typically between 70% and 80% strain. (See Figure 1C for sample stress–strain curves (*n* = 4 to 6 for each condition)).

#### Compositional analysis of infarcted myocardium

Compositional changes to the decellularized infarcted myocardium were assessed with a variety of methods. Total collagen in the infarct region was assessed by a Total Collagen Assay (QuickZyme Biosciences, Voorhout, The Netherlands). In brief, the infarcted region of the decellularized ECM was frozen overnight at −20°C and then lyophilized for 24 hours. Dry tissue was weighed and hydrolyzed in 12 *M* HCl for 20 hours at 95°C. Dilutions of hydrolyzed samples were prepared to obtain absorbance measurements within the range of the standard curve, according to the instructions on the kit (*n* = 3 for each condition). Samples were also prepared for liquid chromatography–tandem mass spectroscopy (LC-MS/MS) through a urea digestion at 4°C with constant agitation via a stir bar after lyophilization for 48 hours. Protein was collected through an acetone precipitation and frozen until samples were sent to the Beth Israel Deaconess Medical Center Mass Spectroscopy Core Facility for further analysis. Resulting spectrum counts were analyzed (*n* = 2 for each condition).

### Differentiation studies

#### ECM solubilization

ECM was isolated from decellularized, healthy, and infarcted hearts at 1 and 4-week time points, as described previously. ECM was perfused with 50 ml diH_2_0 after decellularization with 1% SDS. Whole decellularized hearts were then washed with 50 ml of 0.5% triton X and rinsed again with diH_2_0. Hearts were then washed with 1× PBS through perfusion with a peristaltic pump for 72 hours. PBS was changed every 12 hours. The scar region of the decellularized myocardium was minced and frozen at −20°C overnight. Samples were lyophilized, and dry weight measured. Tissue was solubilized as described previously [41,42]. In brief, matrix was solubilized in a 1 mg/ml solution of pepsin in 0.1 *M* HCl to reach a final solubilized ECM concentration of 10 mg/ml.

#### Glass-slide activation and polyacrylamide (PA) gel formation

To create binding sites for the PA gels, 22 mm × 22 mm glass cover slips were activated by following previously described protocols [43]. Slides were passed over an open flame and smeared with 0.1*M* NaOH, followed by 3-aminopropyltrimethoxysilane. Slides were then transferred to six-well plates and washed with diH_2_0 on an orbital shaker. Water was aspirated, and glass cover slips were incubated in 0.5% glutaraldehyde for 30 minutes. Glutaraldehyde was removed, and slips were washed in diH_2_O for three 5-minute washes. Activated glass cover slips were stored in diH_2_O at 4°C for up to 4 weeks or until use.

PA gels were created at two different stiffnesses (25 and 40 kPa), consistent with previously collected mechanical data; corresponding to healthy myocardium and infarct myocardium after CF remodeling by changing the amount of cross-linking between acrylamide and bis-acrylamide. Gels of 25 kPa stiffness were generated with 10% acrylamide and 0.1% bis-acrylamide, whereas 40 kPa gels were created with 10% acrylamide and 0.2% bis-acrylamide. Gel stiffness was confirmed through mechanical testing with the custom-built uniaxial mechanical stretcher described previously. 400 μg of ECM from each time point was cross linked into 500 μL of PA gel solution of both stiffnesses (25 or 40 kPa) by using *N*-hydroxysuccinimide (NHS) ester to create covalent linkages between amine groups. Rat-tail collagen I (BD Biosciences, San Jose, CA, USA) was incorporated into gels to function as a control protein. HCl was added to the gel to lower the pH to 6.6 to prevent the hydrolysis of NHS. Cross-linking of the acrylamide to bis-acrylamide was achieved with the final incorporation of TEMED and 10% ammonium persulfate (APS).

Activated coverslips were dried in a sterile cell-culture hood, and 30 μl of each gel solution was cast onto a glass coverslip. A nonactivated coverslip was then placed on top of the gel solution to promote polymerization and create an even gel surface. Gels were allowed to polymerize for 30 minutes, and then the nonactivated glass coverslip was removed with a razor blade. Gels were transferred to sterile six-well plates and washed with sterile 1× PBS 3 times for 5 minutes.

#### Cell culture

Primary isolated rat mesenchymal stem cells (rMSCs) were purchased from Cell Applications (San Diego, CA, USA) and cultured in maintenance medium containing 15% FBS in αMEM with 1% Pen-Strep and 2% L-glutamine. Cells were passaged at 80% confluence, and only cells between passages 3 and 6 were included in this study. 30,000 cells were seeded onto each gel and cultured in 20% oxygen in maintenance medium. Cells were also cultured on TCP as a negative control. 24 hours after seeding, gels were transferred to new sterile six-well plates to minimize paracrine signaling between cells seeded on the TCP and cells on gels. Cells were fed every other day and analyzed after 1 week in culture through histology and Western blotting.

#### Histology

Cells cultured on polyacrylamide gels were fixed on day 7 in methanol for 10 minutes at 4°C on an orbital shaker. Cell membranes were permeated through a treatment with 0.05% triton-X for 5 minutes and then rinsed 3 times for 5 minutes in 1× PBS. Samples were blocked in a 5% donkey serum and 0.1% BSA solution in 1× PBS for 1 hour at RT. Primary antibodies for Nkx2.5 and GATA4 (sc-14033 and sc-25310, respectively; Santa-Cruz) were diluted 1:200 in a 0.1% BSA solution. Cells were incubated in the primary solution for 1 hour at RT and then rinsed 3 times for 5 minutes. Secondary antibodies (Alexa Fluor 488-conjucated donkey anti-rabbit 715-545-152, Cy3-conjugated donkey anti-mouse 715-165-150; Jackson ImmunoResearch, West Grove, PA, USA) were diluted at 1:400 in 0.1% BSA solution in PBS. Cells were incubated in the secondary solution for 1 hour at RT. After three 5-minute rinses in PBS, cells’ nuclei were stained with a 1:10,000 dilution of Hoescht fluorescent dye for 5 minutes (Hoescht 33258; Invitrogen). Samples were washed in PBS (3 × 5 min) and then imaged with an Olympus IX 81 fluorescent microscope. In brief, gels were inverted on a glass slide, and two representative images were acquired per condition.

#### Protein isolation and quantification

To quantify the differentiation capacity of the cells within the infarct environments, cells were treated with 0.05% trypsin for 5 minutes and then quenched with fetal bovine serum. Samples were collected via centrifugation at 500 rpm. Supernatant was removed, and pellets were washed with 1× PBS. Samples were collected after a second 500 rpm centrifugation, and pellets were resuspended in ice cold cell lysis buffer consisting of NP40, 40× sodium deoxycholate, sodium orthovanadate, aprotinin, pepstatin, leupeptin, and diH_2_O, as previously described [44]. Samples were sonicated on ice for 20 seconds at 30% amplitude, rotated end over end on a rotisserie for 15 minutes at 4°C and then centrifuged at 13,000 rpm for 15 minutes at 4°C. Total protein was quantified with a Pierce BCA assay.

#### Western blotting

Based on the BCA protein assay, the lanes of 4% to 15% gradient gels (456–1083; Biorad, Hercules, CA, USA) were loaded equally with protein from each condition (typically ranging between 5 and 15 μg). Protein samples were mixed with sample buffer and dithiothreitol and placed on a heat block at 95°C for 5 minutes. Samples were vortexed and briefly centrifuged before loading. Gels were run at 100 V/gel for approximately 35 minutes until the dye front reached the edge of the gel. Protein was transferred onto a nitrocellulose membrane at maximum current (400 mA) for 2 hours. Blots were blocked in 5% milk in TBST (Tris-buffered saline and 10% Tween 20) and probed for Nkx2.5 (SAB2101601; Sigma-Aldrich) and GATA4 (sc-25310; Santa Cruz). Samples were incubated in a 1:400 primary antibody dilution for at least 1 hour at room temperature. Blots were rinsed 3 times for 5 minutes in TBST before incubation in a 1:1,000 dilution of secondary HRP-conjugated antibody (715-035-150 and 711-035-152; Jackson ImmunoResearch, West Grove, PA, USA). After three 5 minute rinses in TBST, blots were developed with enhanced chemiluminescence (ECL) reagents on G: Box Chemi XR5 (Syngene, Cambridge, UK). Expression of the cardiac transcription factors was normalized to cellular β-actin expression (primary 1:1,000 (A5316; Sigma-Aldrich) and secondary 1:5,000 (715-035-150, Jackson ImmunoResearch)). Band intensities were quantified with ImageJ software (NIH, Bethesda, MD, USA) (*n* = 5 for each condition).

### Paracrine secretome studies

Healthy and infarcted matrix of respective time points were solubilized and adsorbed onto 24-well TCP plates at a density of 50 μg/cm^2^. Rat tail collagen I and human plasma fibronectin (Millipore, Billerica, MA, USA) were used as control matrix proteins and adsorbed at the same density. Matrix was diluted in DMEM, applied to wells, and allowed to dry overnight in a sterile biological hood. After three 5 minute rinses with 1× PBS, 500,000 MSCs were seeded per well in 10% fetal bovine serum and 1% Pen-Strep in IMDM. Then 24 hours after seeding, media was changed to a serum and antibiotic-free condition consisting of only IMDM. After 24 hours, cells were isolated for quantitative PCR, and media was collected, centrifuged at 1,000 rpm for 5 minutes, and stored at −80°C for ELISAs and conditioned media studies.

#### Quantitative PCR

Cells were isolated with 0.05% trypsin for 5 minutes and then quenched with fetal bovine serum. Samples were collected via centrifugation at 500 rpm. Supernatant was removed, and pellets were washed with 1× PBS. Samples were collected after a second 500 rpm centrifugation, and RNA was isolated with the RNAeasy kit (74104, Qiagen). Extracted RNA was quantified, and 500 ng was treated with Genomic DNA Elimination mix (Qiagen) and reverse transcribed into cDNA with the Qiagen RT^2^ First Strand Kit in a thermocycler. The two-step reaction consisted of 15 minutes at 42°C followed by 5 minutes at 95°C. The 20 μl reverse-transcription reaction was diluted in 91 μl of nucleotide-free water. Then 17 μl of the diluted reaction was combined with 225 μl of RT2 SYBR Green ROX qPCR Mastermix from Qiagen and diluted with nuclease-free water to reach a final volume of 450 μl. 25 μl of each reaction was applied to the designated wells of a Custom PCR array ordered through SABiosciences, a Qiagen Company. The array was designed to interrogate the expression of 11 genes implicated for their ability to restore function to the infarct through paracrine signaling when expressed by MSCs and included *vegfa, fgf2, pgf, pdgfb, hgf, igf1, tnf, il10, tgfbr2, cxcl12* and *akt1*.

In addition, the array plate contained two housekeeping genes, β-actin and β_2_-microglobulin, as well as three internal controls to assess for genomic DNA contamination, PCR, and reverse transcription efficiency. The array plates are designed to assay six biologic samples for all 16 markers simultaneously. Real-time PCR reactions were carried out on a Stratagene Mx3000P thermocycler in three segments. The first segment consisted of a single cycle performed at 95°C for 10 minutes. Segment 2 consisted of 40 subsequent cycles beginning with 15 seconds at 95°C, followed by 1 minute at 60°C, and ending with fluorescence data collection. Last, segment 3 was carried out for melting curve analysis and consisted of a single cycle at 95°C for 1 minute, followed by 55°C for 30 seconds, fluorescence data collection, and ending with 30 seconds at 95°C.

Primer specificity was verified by a single dissociation curve achieved for each gene of interest. Ct values were calculated at a threshold fluorescence of 0.075 for all plates. Fold change expression was calculated by using the ∆∆Ct method [39] (*n* = 6 for each condition).

#### Enzyme-linked immunosorbent assays

Conditioned media was thawed on ice, and 50 μl of each sample was analyzed with an HGF ELISA kit (R&D Systems), whereas 100 μl was analyzed with an SDF1 SDF1 ELISA kit (USCN Life Science). Assays were performed according to the manufacturer’s instructions (*n* = 6 for each condition).

#### Conditioned media studies

Neonatal rat cardiomyocytes were isolated through a collagenase digestion of the entire heart followed by preplating for 1 hour to achieve a cardiomyocyte-rich population, as described previously. Cells were seeded on 48-well plates at a density of 50,000 cells/cm^2^ in serum containing media (10% horse serum, 2% fetal bovine serum, 1% pen-step in DMEM) and fed every other day. Then 5 days after seeding, media was changed to a serum-free media (50:50 mixture of DMEM and Ham’s F12 Nutrient Mix, 0.2% BSA (wt/vol) (Sigma), 0.5% insulin–transferrin–selenium-X (Invitrogen), and 1% pen-strep, with 0.1 m*M* ascorbic acid (Sigma)). 24 hours later, media was replaced with a 50:50 mixture of conditioned media and IMDM [45]. Complete IMDM was given as a negative control. 1 hour later, media was spiked with 300 μ*M* H_2_O_2_ for 4 hours [46], and cell death was assessed through a Live/Dead cell viability assay (Invitrogen) (*n* = 6 for each condition).

### Statistics

All results were analyzed with appropriate-sized multiple univariate analysis of variance with Student *t* test *post hoc t* testing, and *P* values less than 0.05 were considered statistically significant. Trends with a *P* value less than 0.1 are also identified.

## Results

### Infarct characterization

MI was successfully induced in male Sprague–Dawley rats, as demonstrated by ventricular free wall thinning in the scar region of the infarcted heart after decellularization (data not shown). The infarcted myocardium appears physically distinct from the noninfarcted region, because the increased density of the ECM makes it appear more opaque (Figure 1A, right), allowing for exclusive analysis of the more significantly remodeled tissue.

At 1 week post-MI, the scar matrix increases significantly (*P* < 0.05). 2 and 4 weeks post-MI, the tissue is significantly stiffer than both the healthy and 1 week infarct matrix (*P* < 0.05) (Figure 1C). This increase in stiffness may be related to a significant increase in the number of small diameter fibers deposited throughout the tissue after MI, as revealed by SHG imaging (Figure 1B). The average image volume backward SHG intensity decreased 25% from 99.9 to 74.6 a.u. between healthy tissue and 1 week after MI, with intensities increasing over the following weeks to an average value of 118.9 at week 4 (19% increase relative to healthy tissue). A much weaker SHG intensity was collected in the forward direction (Figure 1B), which may be affected by light scattering through these thick tissue samples. Interestingly, the average TPEF image intensity was 59% to 67% lower at all postinfarct time points relative to healthy tissue, suggesting fewer elastin or collagen cross-links present in each average volume.

**Figure 2:**
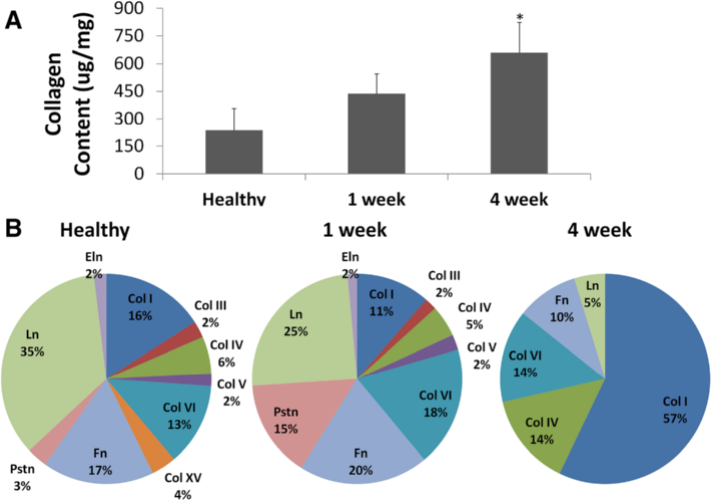
**Characterization of the infarct matrix composition after myocardial infarction. (A)** Total collagen content within the 4-week infarct is significantly greater than both the healthy and 1-week conditions (*n* = 3 for each condition and *P* < 0.05). **(B)** LC-MS/MS spectrum count analysis describes the relative percentages of each matrix protein identified within the decellularized healthy left ventricle or scar. Note that Pstn is periostin, Ln is laminin, Eln is elastin, Fn is fibronectin, and Col is collagen. (*n* = 2 for each condition).

Compositional analysis confirmed that the most significant changes to the ECM composition occur after CF activation (usually thought to occur around 2 weeks after MI). At 1 week after MI, a slight, although not significant increase in total collagen is measured by a total collagen assay. At 4 weeks after MI, the increase in collagen is significant (*P* < 0.05) (Figure 2A). Spectrum-count analysis of LC-MS/MS data revealed dramatic alterations in protein composition after MI (Figure 2B). In the healthy heart, laminin, fibronectin, and collagen I comprise nearly 70% of the total protein within the heart. However, the complexity of the composition is highlighted by the presence of periostin, elastin, collagen III, collagen V, and other collagen isoforms. At 1 week after MI, there are noticeable alterations in the composition of the scar. In particular, periostin expression increases nearly fivefold, as well as slight increases in fibronectin and collagen VI. While total elastin content remains the same, there is a decrease in laminin and collagen I expression. In general, the matrix is composed of relatively similar proportions of total collagen (43% collagen in healthy heart versus 38% at 1 week after MI). The most significant alterations in composition are observed at the 4 week time point. Collagen I comprises 57% of all matrix proteins, and 85% of the composition is represented by total collagen. Fibronectin and laminin represent the other 15% of the composition. These results demonstrate the dynamic remodeling process that occurs after MI.

### Generation of *in vitro* cell-culture platform

**Figure 3:**
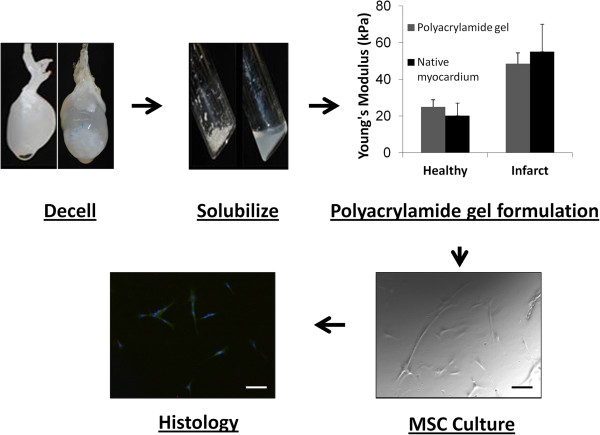
**Schematic of the development of an *in vitro *cell-culture platform to characterize MSC fate within infarct microenvironment.** Whole hearts isolated from both healthy and infarcted (1- and 4-week time points) animals are decellularized, solubilized, and incorporated into polyacrylamide gels of stiffnesses corresponding to both healthy and infarcted myocardium. Histologic analysis reveals that cells attach and spread along the gels (scale bar is 100 μm).

Polyacrylamide gels were mechanically tested to confirm the stiffness corresponded to both healthy and diseased myocardium (Figure 3). The incorporation of solubilized ECM of both healthy and infarcted hearts into the gels provided binding sites for MSCs. Histologic analysis confirmed the ability of cells to adhere to and survive on the gels for more than 1 week (Figure 3).

### Assessment of MSC differentiation

**Figure 4:**
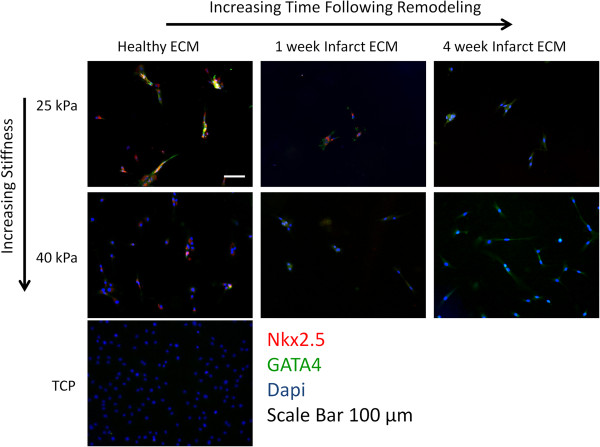
**Early cardiac differentiation is abrogated by the infarct microenvironment.** Representative histologic images of MSCs cultured on polyacrylamide gels modeling the various infarct environments of our cell-culture system. Nkx2.5 is red, GATA4 is green, and Dapi nuclear stain is blue. Scale bar is 100 μm.

Histologic analysis reveals that MSCs cultured in the gel containing healthy, decellularized cardiac ECM at a physiologically relevant stiffness (25 kPa) express the early cardiac transcription factors, Nkx2.5 and GATA4 (Figure 4). However, a significant decrease in the expression of these markers was observed on gels of higher stiffness (40 kPa). Note that Nkx2.5 expression is primarily in the nucleus, with diffuse staining in the cytoplasm. Similarly, cells cultured on gels of decellularized infarct ECM isolated 1 week after induction of an infarct expressed only Nkx2.5 and GATA4 robustly on gels of a 25 kPa stiffness, whereas expression was minimal on gels of 40 kPa stiffness. Limited Nkx2.5 expression was observed when cells were cultured on gels with 4 week matrix of either stiffness. In contrast, GATA4 expression appeared robust on gels of increased stiffness containing 4 week infarct matrix. Minimal expression of either marker was observed on MSCs cultured on tissue culture plastic.

**Figure 5:**
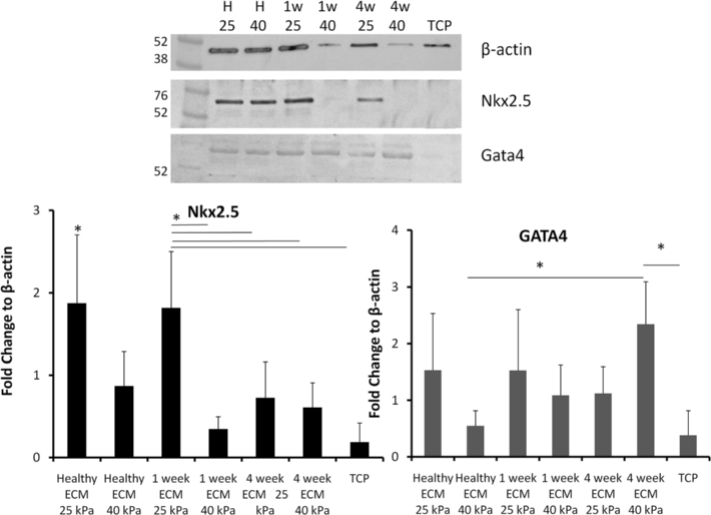
**Protein expression of early cardiac transcription factors is influenced by infarct microenvironment.** Representative Western blot images for both cardiac transcription factors (Nkx2.5 and Gata4) as well as a housekeeping gene (β-actin) are presented. Relative expression levels for each cardiac marker are normalized to β-actin and presented for each condition. For the Nkx2.5 plot, the healthy, 25-kPa condition is significantly higher than all other conditions. For GATA4, the 4 week, 40 kPa condition is significantly greater than the TCP condition and thehealthy, 40 kPa gel condition (*P < 0.05 and *n* = 5 for each condition).

Western blot analysis of total cell lysate revealed similar trends regarding the influence of biophysical cues of the infarct on the expression of cardiac markers (Figure 5). The most robust expression of Nkx2.5 was observed by cells cultured on gels of 25 kPa stiffness with healthy and 1 week infarct ECM, as compared with all other conditions (*P* < 0.05) (Figure 5). Cells cultured on 4 week matrix on gels of either stiffness did not express significantly more Nkx2.5 than cells cultured on TCP. Conversely, the 4 week infarct ECM significantly promoted the expression of GATA4 on gels of 40 kPa stiffness, as compared with both healthy and the control, TCP (*P* < 0.05) (Figure 5). However, no significant difference in GATA4 expression was seen when gels were cultured on 4 week matrix of a lower, 25 kPa stiffness. We performed a preliminary study with gels incorporated with Collagen I and did not see significant expression of Nkx2.5 or GATA4 through immunohistochemistry. This was further confirmed through Western blotting (see Additional file 1: Figure S1).

### Assessment of MSC secretome as a function of matrix remodeling after I

**Figure 6:**
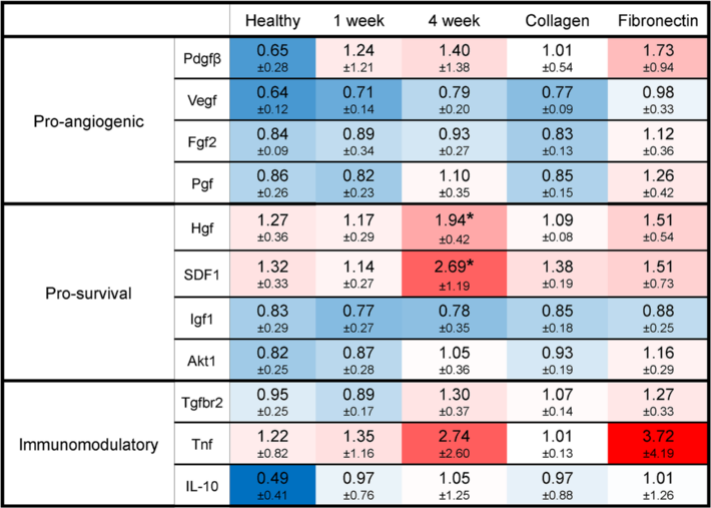
**Evaluation of MSC secretome as a function of matrix composition through mRNA expression.** The average fold change in mRNA expression ± the standard deviation for proangiogenic, prosurvival, and immunomodulatory factors is calculated relative to cells cultured on TCP. The data are averaged over multiple matrix isolations (*n* = 6). The color scaling of each cell denotes the degree to which the expression is upregulated (red intensity), downregulated (blue intensity), or maintained constant (white). The conditions in which the growth factor expression is significantly upregulated are denoted by an asterisk.

**Figure 7:**
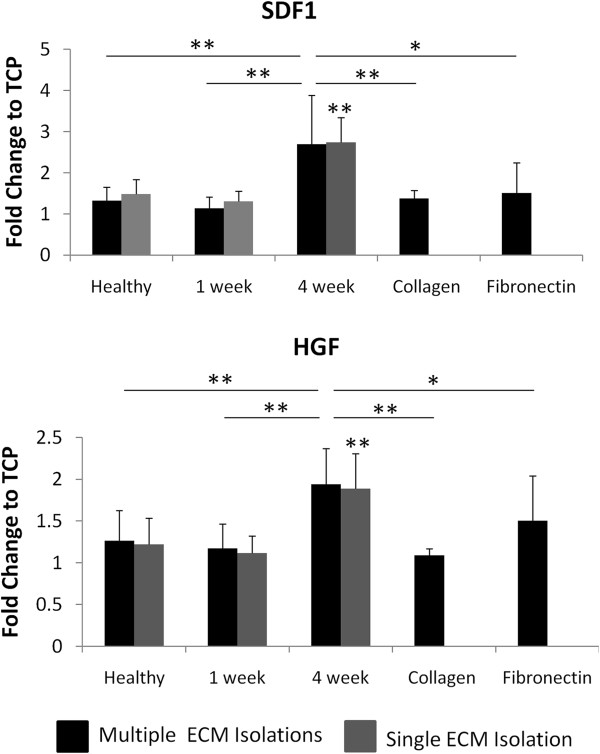
**The mRNA expression of prosurvival growth factors is modulated by matrix composition.** The fold change in mRNA expression for *cxcl12* and *HGF* is calculated relative to cells cultured on TCP. The data are presented for both a single ECM isolation (*n* = 3) and data averaged over multiple matrix isolations (*n* = 6). For both genes, the 4 week time point has significantly higher expression than either the healthy or 1 week condition. A single * represents *P* < 0.1, and ** represents *P* < 0.05.

**Figure 8:**
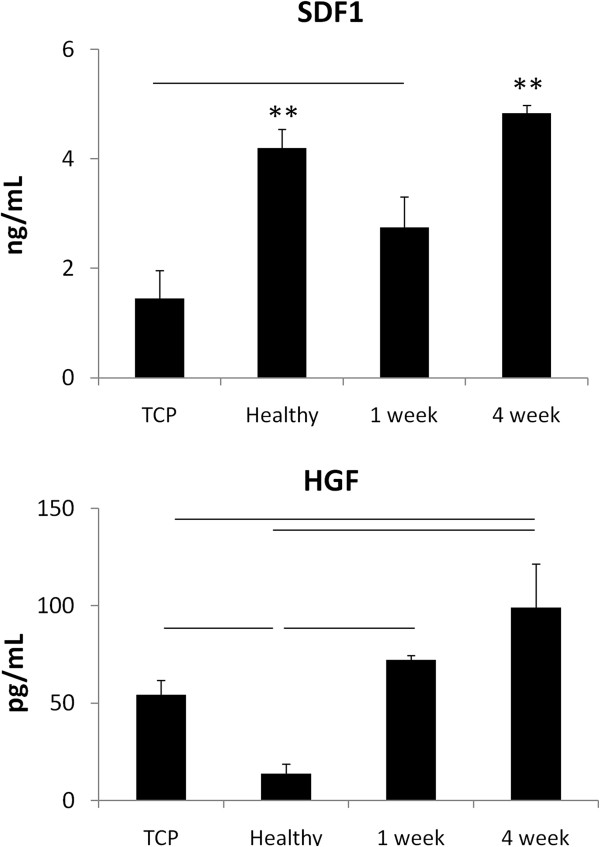
**SDF1 and HGF concentrations in conditioned media samples are modulated as a function of substrate composition.** SDF1 is present at the highest concentration in conditioned media derived from MSCs cultured on healthy and 4 week matrix (*P* < 0.05). However, cells cultured on 1 week matrix secrete more SDF1 into the media than do those cultured on TCP (*n* = 3). The concentration of HGF in conditioned media samples derived from cells cultured on 4-week matrix is significantly higher than healthy matrix or TCP (*P* < 0.05). Cells cultured on healthy matrix secrete significantly less HGF than those under all other conditions (*P* < 0.05) (*n* = 3).

**Figure 9:**
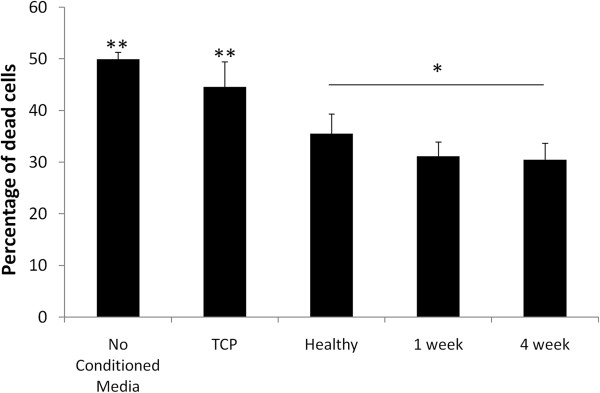
**MSC-conditioned media promotes cardiomyocyte survival after oxidative stress.** The conditioned-media samples derived from MSCs cultured in the presence of matrix have a greater ability to rescue oxidatively stressed cardiomyocytes as compared with those cells cultured on TCP (*P* < 0.05) or media that has not been conditioned by MSCs (*P* < 0.05). Conditioned media samples from cells cultured on 4 week matrix have a greater ability to prevent cardiomyocyte death as compared with cells cultured on healthy matrix (*P* < 0.1) (*n* = 6).

Of the soluble factors assayed, *hgf* and *cxcl12* expression were both significantly altered as a function of matrix composition (Figure 6). The expression of both prosurvival growth factors was greatest in the presence of the chronic infarct matrix. Although the expression of proangiogenic (*pdgfb, vegfa, fgf2,* and *pgf*) and immunomodulatory (*tgfbr2* and *il10*) factors were also elevated in the presence of the 4 week matrix, as compared with both the healthy and 1 week matrices, these trends did not maintain significance across multiple matrix isolations because of the inherent variability associated with matrix remodeling (Figure 6). However, the enhanced expression levels of *cxcl12* and *hgf* were upheld across both single and multiple matrix isolations (Figure 7). To verify that alterations in gene expression affected functional outcomes, we looked at the release of HGF and SDF1 by MSCs in the presence of healthy, 1 week, and 4 week ECM, each derived from a single isolation. ELISAs demonstrated that both growth factors were present in the conditioned media at the greatest concentration when cells were cultured on 4 week matrix (*P* < 0.05). However, it appears that the healthy matrix inhibits the production and release of SDF1, as compared with the 1-week matrix or TCP (*P* < 0.05) (Figure 8). Alternatively, HGF is down-regulated on the 1 week matrix as compared to the healthy. However, all of the three matrices promote HGF secretion compared with TCP (*P* < 0.05) (Figure 8). This increase in prosurvival growth factors in the media of cells cultured on healthy, short term, and long term infarcted matrix was able to rescue oxidatively stressed cardiomyocytes *in vitro* as compared with those cells cultured on TCP (Figure 9).

## Discussion

Extensive research has demonstrated how the extracellular environment plays a critical role in regulating cellular, tissue, and whole-organ physiology [19-21]. Therefore, it is necessary to understand how alterations in the physical and chemical properties of the ECM change during disease progression and how these changes ultimately influence resident cells and the potential of therapeutic strategies to regenerate native tissue function. This study is the first to use decellularization to study alterations in the extracellular matrix of diseased tissue, and the results described herein demonstrate how the altered matrix affects the potential for therapeutic intervention.

Analysis of the decellularized infarct tissue revealed significant alterations in both the mechanical and compositional properties of the ECM with remodeling time after MI. Stiffness measurements of the decellularized infarct followed trends similar to those derived from native, cellular scar tissue [33,47]. In general, infarct ECM stiffness increased slightly immediately after infarction (within the first week of remodeling), but the most dramatic alterations in stiffness occurred during the later stages of remodeling after CF activation, with a nearly threefold increase in stiffness (2 and 4 weeks after MI). However, it is important to note that the magnitude of stiffness values varies greatly between native infarcted tissue and decellularized infarcts. As previously reported, decellularized tissues have mechanical properties that are distinct from their native form [29], because the absence of cells increases the density of the ECM, thereby increasing the overall apparent stiffness. In our cell-culture platform, we chose to recapitulate the stiffness of the cellularized infarct because it is more characteristic of the microenvironment seen *in vivo* by implanted cells, although investigations into the effects of higher stiffness may be warranted in the future.

Assessment of collagen fibril content and alignment via SHG of decellularized infarcts allows a unique measure of the dynamic changes in the organizational patterns of collagen fibers as a function of time after MI. Our imaging analysis confirmed earlier findings that elucidated that the majority of all collagen fibers within the scar are highly aligned [48]. Although the measured SHG intensities within the image volume can be affected by changes in the light-scattering properties of the tissue and/or the microstructural organization of collagen fibrils within the larger fiber bundles [49], a trend of increasing SHG image intensity with post-MI time points further supports evidence of increased collagen deposition over time. Collectively, the compositional analysis and SHG imaging of decellularized tissue demonstrate increased collagen deposition and organization into aligned fiber bundles after infarct.

Alterations in scar mechanics are likely a result of the changing composition and structural organization of the matrix. Our findings are in accordance with previous work [50] demonstrating an increase in total collagen deposition after infarction, which serves to stabilize the injured organ. Our work confirms the earlier finding that the 4 week infarct contains more collagen than in either the healthy or 1 week conditions [50]. LC-MS/MS analysis further confirmed this assessment, as the composition of the infarct at 4 weeks after MI consists nearly entirely of collagen, whereas the healthy and 1 week matrix is more diverse and composed of a variety of matrix proteins.

It is important to note that this method of analysis has a limited potential for identifying those proteins that are present in relatively low abundance, because higher-abundance proteins will comprise the majority of all spectrum counts. In the 1 week infarct matrix, we observed a decrease in collagen I, laminin, collagen IV, and collagen XV. Phatharajaree *et al*. previously reported an increase in MMP expression within 2 days and maximal expression by 7 days after coronary artery ligation [51], which could explain decreases in ECM protein content. Although others have reported an increase in laminin [52], collagen I [53], collagen III [53], and collagen IV [54] gene expression immediately after MI, it is important to note the deposition of functional protein occurs several days after the transcriptional activation of genes [55]. Collagen XV is involved in matrix organization within the heart, and its deficiency results in an increased sensitivity to cardiac stress [56]. Its absence within the LC-MS/MS spectrums derived from infarcted hearts supports our observation of disorganized fibers via SHG imaging as early as 1 week post-MI.

We also show an increase in periostin, fibronectin, and collagen XI within 1 week of artery ligation. Periostin is critical for stabilizing the ventricle wall after infarction, and our findings support previous work, which demonstrates that periostin expression is induced after myocardial ischemia [57]. Other work has also demonstrated a rapid increase in fibronectin [58] and collagen VI expression [52] immediately after MI. Although fibronectin has been identified for its beneficial role in wound healing [59], collagen VI negatively affects cardiac function after MI (through increased cardiomyocyte apoptosis and fibrosis, as compared with collagen VI-deficient mice) [60].

The most dramatic alterations in protein content are observed 4 weeks after coronary artery ligation with an absence of several critical cardiac matrix proteins, including periostin, elastin, collagen III, collagen V, and collagen XV. These results suggest that the scar has been negatively remodeled by 4 weeks because of the lack of elastin [61] and collagen V [62]. The deposition of newly synthesized matrix proteins is likely disorganized, given the absence of collagen XV [56]. Overall, the remodeled matrix is dominated by collagen content, which increases the stiffness of the organ and minimizes its capacity to function normally [63].

The striking difference between the remodeled and native tissue is further illustrated by the capacity of the different ECM to elicit early cardiac differentiation in MSCs. The composition and stiffness of healthy myocardium promoted early cardiac differentiation, as evidenced by an increase in Nkx2.5 and GATA4 expression, as compared with TCP. Although the early infarct matrix at 1 week did not negatively influence the expression of either transcription factor, the increased stiffness characteristic of the infarct significantly abrogated the differentiation capacity of the cells. These results support previous findings by Engler *et al*. [23], which demonstrated that MSCs have a greater capacity for myogenic differentiation on polyacrylamide gels of lower stiffness (10 kPa) as compared with those of higher stiffness (100 kPa). In addition, Tan *et al*. demonstrated that MSCs cultured on adsorbed collagen V upregulated their expression of both Nkx2.5 and GATA4, as compared with collagen I [22]. This follows our finding, which demonstrated similar expression levels of Nkx2.5 on gels with healthy and 1 week matrix, which both contain similar abundances of collagen V. It is important to note that the more significantly remodeled matrix of the 4 week time point drastically altered the expression of both transcription factors. Although Nkx2.5 expression is negligible on either stiffness in the presence of 4 week matrix, GATA4 expression is dramatically enhanced by this matrix on the gel of increased stiffness (40 kPa). This suggests that complex interactions occur between stiffness and composition, which regulate MSC differentiation. Further experiments are needed to identify which individual peptides or proteins may be promoting or inhibiting cardiac differentiation within the ECM of the 4 week infarct. By identifying additional matrix proteins that influence cellular differentiation, we may be able to manipulate the extracellular environment *in vivo* to enhance cellular differentiation and ultimately improve myocardial regeneration.

Whereas literature provides conflicting evidence demonstrating both an ability [37] and inability [36] of implanted MSCs to differentiate toward a cardiomyocyte lineage, significant work has illustrated an ability of MSCs to express cardiac specific markers including Nkx2.5, GATA4, and α-actin within the infarct environment [35,64]. In particular, Quevedo *et al*. [34] observed some capacity for cardiac differentiation when cells were implanted in chronic cases of MI as compared with acute intervention. These findings are in agreement with our system that identified that the composition and stiffness of the later infarct environment was promoting MSC expression of GATA4 as compared with the stiffness and composition characteristic of the 1-week time point. However, it is important to note that the MSCs within our gel system did not express later cardiac transcription factors, including Mef2c and Tbx5 (data not shown), and were unable to differentiate into mature cardiomyocytes.

Despite their limited potential for cardiac differentiation, MSCs have still demonstrated an ability to restore some minimal, although statistically significant, function to the heart after MI during clinical trials, through the presumed mechanism of paracrine signaling [11,12,65]. Therefore, we sought to investigate whether the release of paracrine signals by MSCs is influenced by remodeling time after MI. Previous work by Li *et al.* demonstrated that the overexpression of GATA4 by MSCs enhanced the therapeutic potential of these cells by increasing their expression of particular growth factors within the infarct environment [66]. In particular, proangiogenic growth factors are capable of improving left ventricle function by increasing capillary density within the scar and border zones of infarcted hearts when secreted by MSCs *in vivo*[13]. These factors include VEGF [67], PGF [68], FGF2 [68], SDF1 [69], and HGF [70]. All five of these factors demonstrated increased expression by MSCs when cultured on the 4-week matrix (Figures 6 and 7), which suggests that the composition of the chronically remodeled heart enhances the ability of these cells to promote angiogenesis within the infarct.

In addition to their roles in angiogenesis, HGF and SDF1 are recognized for their anti-fibrotic and cytoprotective roles within the injured myocardium. HGF signaling is known to be integrin mediated [71] (the HGF receptor, C-met, physically interacts with integrins and together they regulate downstream processes [72]), but research has also demonstrated that the overexpression of SDF1 leads to enhanced HGF signaling. Given that our results reveal similar trends for both growth factors (increased expression on 4 week matrix), it is unclear whether we are observing an independent or combinatorial effect. Further investigation is needed, but it is important to note that AKT1 expression was also slightly elevated on the 4-week matrix (Figure 6). AKT overexpression has been shown to promote the release of paracrine signals by MSCs (thereby increasing the benefit achieved after implantation) [36], and its activation is integrin mediated [73]. Therefore, it is possible that the expression of AKT1 is altered by matrix composition and accounting, at least in part, for to the alterations in growth factor secretion observed. Functional tests demonstrated that the upregulation of SDF1 and HGF expression and secretion translated to improved survival for stressed cardiomyocytes *in vitro*.

Although the direct mechanism by which the matrix composition is influencing growth factor expression and secretion is unclear, earlier work has demonstrated that the differentiation potential of MSCs toward bone and tendon lineages is driven by their paracrine profile, which is modulated by matrix composition [74]. Therefore, it follows that the release of paracrine signals within the infarct environment is likely altered as a function of the remodeling time (which dramatically augments the composition of the matrix). The 4 week matrix has the most striking alterations in composition and therefore has the largest impact on paracrine signaling by MSCs. 

In addition, the bioavailability of growth factors within the microenvironment may itself be influenced by the matrix composition. Earlier work demonstrated that VEGF has enhanced biologic activity on fibronectin and vitronectin substrates, whereas PDGF has increased affinity for collagen substrates of various isoforms [75,76]. Therefore, it is possible that once the MSCs secrete these soluble factors, they are maintained within the infarct matrix at varying affinities and bioavailabilities as a function of matrix composition [77].

Last, the cellular response to growth-factor stimulation is known to be integrin dependent. Cardiomyocyte proliferation achieved via heparin-binding EGF-like growth factor is dependent on β1-integrin stimulation. This suggests a dynamic cross-talk between integrin expression and growth factor stimulation [27]. Although further work is needed to identify which of these mechanisms is responsible for the measured effect of matrix composition on soluble factor expression by MSCs, it is clear that the therapeutic potential of these cells is enhanced within the extracellular environment of the remodeled infarct.

Although investigation into the therapeutic potential of MSCs within the acutely infarcted heart is more common [78], several investigators have observed significant repair by MSCs when injected 1 to 3 months after infarction [78-81]. For example, Miyahara *et al.*[80] demonstrated that the implantation of MSCs 4 weeks after coronary artery ligation promoted angiogenesis, reversed wall thinning, and improved left ventricular function [80]. These studies support our finding that MSCs maintain the potential to initiate significant repair against a chronic infarct. It is also possible that the therapeutic potential of these cells in the acute infarct will be enhanced after co-injection with matrix proteins that are representative of the 4 week time point. Earlier work demonstrated that the injection of decellularized porcine ventricular ECM promoted angiogenesis via enhanced arteriole formation [32], and we believe the co-injection of matrix and MSCs will only further enhance this therapeutic strategy.

## Conclusions

This study demonstrates a novel mechanism by which the extracellular environment of the infarct regulates the therapeutic potential of MSCs. By specifically isolating and characterizing the diseased matrix, we were able to understand both its positive and negative influence on cell therapy applications. Further work with this cell culture system has the potential to increase both the efficiency and efficacy of cell therapy treatment of MI, to ultimately reduce the prevalence and severity of HF.

## Abbreviations

AKT1: Protein kinase B; APS: ammonium persulfate; CF: cardiac fibroblast; CVD: cardiovascular disease; ECL: enhanced chemiluminescence; ECM: extracellular matrix; FGF2: fibroblast growth factor 2; HF: heart failure; HGF: hepatocyte growth factor; IGF: insulin-like growth factor; IL-10: interleukin 10; LC-MS/MS: liquid chromatography–tandem mass spectroscopy; MI: myocardial infarction; MSC: mesenchymal stem cell; NHS: *N*-hydroxysuccinimide; PA: polyacrylamide; PBS: phosphate-buffered saline; PDGF: platelet-derived growth factor β; PGF: placental growth factor; SDF1: stromal cell-derived factor 1; SDS: sodium dodecylsulfate; SHG: second harmonic generation; TBST: Tris-buffered saline and 10% tween 20; TCP: tissue culture plastic; TGF-βR2: transforming growth factor-β receptor II; TNF-α: tumor necrosis factor-α; TPEF: two-photon excited fluorescence; VEGF: vascular endothelial growth factor.

## Competing interests

The authors declare that they have no competing interests.

## Authors’ contributions

KS carried out the *in vivo* animal work, characterized the alterations to decellularized matrix composition and mechanics as a function of remodeling time, developed the *in vitro* cell-culture platform and characterized cell fate through protein and gene-expression assays. KQ acquired and analyzed the SHG images. KT helped to develop the *in vitro* model system and collected preliminary data. IG participated in the design and coordination of the SHG image acquisition and analysis. LB conceived of the study, participated in its design and coordination, and helped to draft the manuscript. All authors read and approved the final manuscript.

## Supplementary Material

Additional file 1: Figure S1 Collagen I-coated polyacrylamide gels elicit minimal expression of cardiac transcription factors. Representative histologic images of MSCs cultured on polyacrylamide gels coated with Collagen I and stained for markers of Nkx2.5 and GATA4. Scale bar is 100 μm. Collagen I-coated gels (*n* = 1) and TCP (*n* = 5) elicit similar levels of expression of Nkx2.5 and Gata4, as demonstrated through Western blot analysis. Click here for file
