# Supplementary figures and images for: Extracellular matrix remodeling following myocardial infarction influences the therapeutic potential of mesenchymal stem cells
Source: Stem Cell Res Ther. 2014 Jan 24;5(1):14. doi: 10.1186/scrt403 (PMC4055039; doi:10.1186/scrt403)

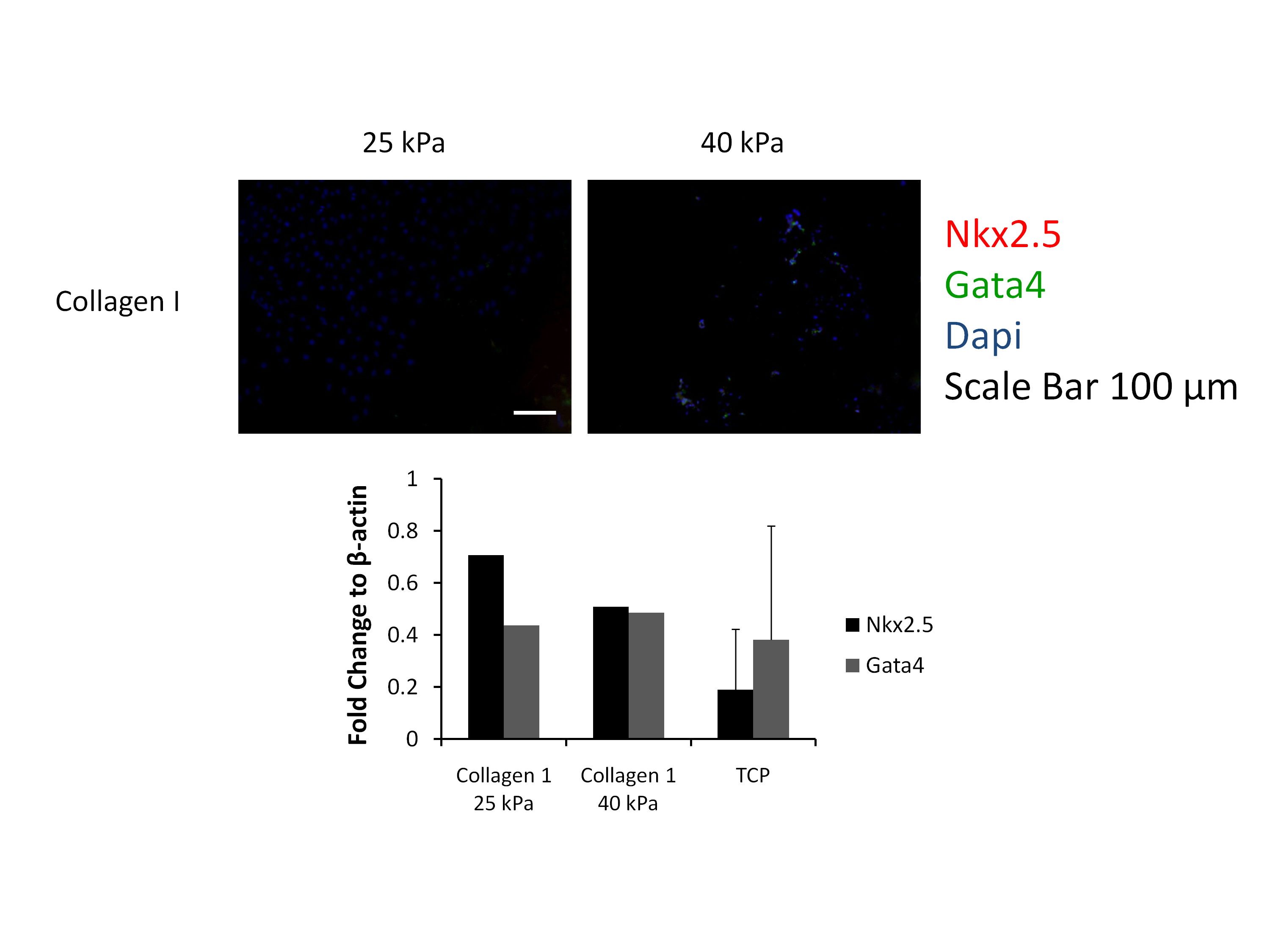

Supplement: Additional file 1: Figure S1 — Collagen I-coated polyacrylamide gels elicit minimal expression of cardiac transcription factors. Representative histologic images of MSCs cultured on polyacrylamide gels coated with Collagen I and stained for markers of Nkx2.5 and GATA4. Scale bar is 100 μm. Collagen I-coated gels (n = 1) and TCP (n = 5) elicit similar levels of expression of Nkx2.5 and Gata4, as demonstrated through Western blot analysis. [file scrt403-S1.tiff]
